# Development and Evaluation of a Novel Pellet-Based Tablet System for Potential Colon Delivery of Budesonide

**DOI:** 10.1155/2012/905191

**Published:** 2012-04-22

**Authors:** Jaleh Varshosaz, Jaber Emami, Naser Tavakoli, Mohsen Minaiyan, Nakisa Rahmani, Farid Dorkoosh

**Affiliations:** ^1^Department of Pharmaceutics, Faculty of Pharmacy and Novel Drug Delivery Systems Research Center, Isfahan University of Medical Sciences, Isfahan 81746-73461, Iran; ^2^Department of Pharmacology, Faculty of Pharmacy and Isfahan Pharmaceutical Sciences Research Center, Isfahan University of Medical Sciences, Isfahan 81746-73461, Iran; ^3^Department of Pharmaceutics, Faculty of Pharmacy, Tehran University of Medical Sciences, Tehran, Iran

## Abstract

Budesonide, a potent glucocorticoid, is used for the treatment of inflammatory bowel diseases. Current available oral formulations of budesonide have low efficacy against ulcerative colitis because of the premature drug release in the upper part of the gastrointestinal tract. In this paper a pH- and time-controlled colon-targeted pellet-based tablet of budesonide was established. Pellet cores were prepared by extrusion-spheronization method and further coated with xanthan gum (barrier layer), Eudragit NE30D and L30D55 combination (inner layer), and Eudragit FS30 (as enteric layer) sequentially to achieve the required release profile. The coated pellets then compressed into tablets using inert tabletting granules of Cellactose or Pearlitol. Release studies, performed in simulated gastric, intestinal, and colon pH were used in sequence to mimic the gastrointestinal transit. The influence of formulation variables like barrier layer thickness, inner layer composition, and enteric coat thickness on drug release were investigated and the coated pellets that contained 12% weight gain in xanthan gum layer, Eudragit L30D55 and Eudragit NE30D with a ratio of 3 : 7 in inner layer with 30% weight gain and 25% weight gain in Eudragit FS layer were found to protect the drug release in stomach and small intestine and 83.35 ± 2.4 of budesonide was released at 24 h. The drug release from the tablets prepared using 40% Cellactose 80 as tableting excipient was found to be closely similar to that of uncompressed pellets.

## 1. Introduction

Controlled-release multiunit dosage forms (e.g., pellets, granules, or microparticles) are becoming more and more important on the pharmaceutical market, as they provide several advantages compared to single-unit dosage forms (e.g., tablets or capsules) [1]. With regard to the final dosage form, the multiunits can be filled into hard gelatin capsules [2] or be compressed into disintegrating tablets [3, 4]. The advantages of tableting multiunits include less difficulty in oesophageal transport, and thus a better patient compliance. Tablets can be prepared at a lower cost because of the higher production rate of tabletting process. The expensive control of capsules integrity after filling is also eliminated. In addition, tablets containing multiunits could be scored without losing the controlled release properties, which allows a more flexible dosing regimen [5].

One challenge in the production of such systems is maintaining the desired drug release after compaction as the application of compaction pressure can lead to structural changes in the film coating and consequently altered drug release [6]. The compression-induced changes in the structure of a film coating may depend on formulation factors such as mechanical properties of the film and incorporated excipients of pellets [7]. Another important parameter that should be considered is choosing of proper cushioning agent to prevent of undesirable fusion of coated pellets by the compression [8]. In this study, a colon delivery formulation of budesonide was designed based on pH and time-dependent approach where film-coated pellets were compressed into multiparticulate tablets. Budesonide, a potent glucocorticoid, is a standard drug for the localized treatment of inflammatory bowel diseases [9]. Current available oral formulations of budesonide have low efficacy against ulcerative colitis (UC) because of the premature drug release in the upper part of the gastrointestinal tract (GIT) [10]. In this study, triple-layer-coated pellets of budesonide were developed for colonic targeting. The pellets were prepared by extrusion/spheronization method and further coated sequentially with various polymers. Then they were compressed into tablets using Cellactose 80 or Pearlitol 200 granules as tabletting excipient. The expected *in vitro *release pattern selected for the colon targeting was no drug release in simulated gastric fluid and not more than 10% of drug release up to the end of small intestine (4 hrs) and more than 80% of drug release up to 24 hrs in the simulated colon.

## 2. Materials and Methods

### 2.1. Materials

Budesonide was obtained as a gift sample from Astra Zeneca (UK). Eudragit FS 30 D, Eudragit NE30D, and Eudragit L30D55 were donated by Evonik Degussa Corporation (Germany). FMC (Ireland) provided the microcrystalline cellulose as Avicel PH 101 and Avicel RC581. Talc and triethyl citrate (TEC) were obtained from Kirsh Pharma (Germany); lactose monohydrate 200 and Cellactose 80 (Coprocessed lactose-cellulose-compound) were obtained from Meggle (Germany). Pearlitol 200 (direct compressible mannitol) was obtained from ROQUETTE (France). Xanthan gum was obtained from Arthur Branwell (UK). All other materials used were of analytical reagent grade and purchased from Merck Co. (Darmstadt, Germany).

### 2.2. Preparation of Pellets by Extrusion/Spheronization

Core pellets containing budesonide (1.5% w/w), Avicel PH 101 (6% w/w), Avicel RC581 (24% w/w) and lactose (68.5% w/w) were prepared by extrusion-spheronization using model 20 extruder and model 250 spheronizer (Caleva, UK). Distilled water was used as granulation liquid. They were dried at room temperature for 24 h. Pellets with the size range of 840–1000 *μ*m were used for subsequent coating.

### 2.3. Preparation of Budesonide-Coated Pellets

**Figure 1:**
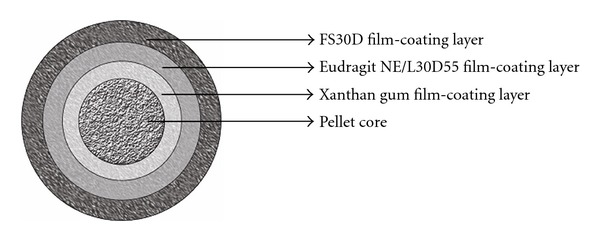
Schematic of the multilayer film coated pellet of Budesonide.

Budesonide containing pellet cores were coated with various polymers (Figure 1) using a top spray fluidized bed coater (VECTOR Corporation, Marion, Iowa) at coating conditions as shown in Table 1.

#### 2.3.1. Inner Coat

A dispersion containing 0.25% w/v of xanthan gum prepared by dispersing gum in 70 : 30 ethanol: water mixture containing plastisizer, triethyl citrate (TEC) (5% w/v, based on amount of solvent).

Before addition of plasticizer, gum mucilage was stirred gently for a period of 10 min with magnetic stirrer and the dispersion was allowed to equilibrate for a period of 3 h. The solution was allowed to stand at room temperature for 24 h.

#### 2.3.2. Middle Coat

Eudragit NE30D and Eudragit L30D55: the mixture of these two polymers (9 : 1, 8 : 2, and 7 : 3 ratio (w/w)) was added to a beaker, placed on a magnetic stir plate and mixed with a slow agitation for a period of 1 h. Talc, equal to 50% (w/w) of the total dry polymer weight and TEC equal to 20% (w/w) of Eudragit L30D55 dry polymer weight were added in a separate volume of water and dispersed via high shear mixing. The dispersion was then added to the former blend of Eudragit dispersion. The resulting dispersion had a total solid content of 15% and was allowed to mix for a further 10 min prior to application to the budesonide pellets.

#### 2.3.3. Outer Coat

Eudragit FS30D aqueous dispersion was diluted twice with water before use. Talc (50% on dry polymer weight) and TEC (10% on dry polymer weight) were added as glidant and plasticizer, respectively. After each coating run, pellets were fluidized for a further 15 min before checking the weight gain and then subsequently cured. A series of coated products were produced with different film thicknesses and quantified by the total weight gain (% TWG).

The compositions of various pellet formulations are shown in Table 2.

### 2.4. Tabletting of Coated Pellets

To produce multiple unit tablets, the optimized batch of coated pellets in size range of 1190–1410 *μ*m were mixed with different ratios and different proportion of the inert tabletting granules. Diluents of Cellactose or Pearlitol granules were used for formulation development. The granules were prepared using wet granulation method. Distilled water (the granulating liquid) was added to Cellactose or Pearlitol and mixed for 10 min to produce a wet mass of suitable consistency, which was passed through 1 mm diameter sieve and were dried in an oven at 50°C. The coated budesonide pellets and inert granules were compressed into tablets using a single punch-tabletting machine fitted with round flat faced 11.6- mm- diameter punches and dies at 70 N compressions to get sufficient strength. The compositions of various tablet formulations are shown in Table 2. Magnesium stearate was used as a lubricant in all formulations (1%). 

### 2.5. *In Vitro* Release Studies for Budesonide Pellets and Tablets

The dissolution performance of the coated pellets and prepared tablets was tested using USP method 2 (rotating paddle at 50 rpm, 37 ± 0.5°C, *n* = 6). For the first two hours of the test, 0.1 N HCl (pH 1.2) (250 mL) was used as the test medium. After two hours, the test medium was changed to phosphate buffer solution (PBS) pH 7.4 (250 mL) for four hours and finally to PBS pH 6.8 (250 mL) for 18 h. In all drug release studies, 0.5 percent (w/v) of sodium lauryl sulphate was used in each dissolution medium to maintain sink conditions [11]. Samples were withdrawn from the dissolution vessels at regular intervals and the concentration of budesonide in solution was monitored by HPLC method described below [12]. All tests were done on 6 tablets of each formulation and the mean of results was considered in release profiles.

### 2.6. Budesonide Analysis

The quantitative determination of budesonide in assay and dissolution studies was performed by HPLC method equipped with UV detector using dexamethasone as an internal standard. The analysis was carried out by using a Shimpack C8 column (150 mm × 4.6 mm, 5 mm particle size) at a wavelength of 244 nm. The mobile phase consisted of acetonitrile, monobasic potassium phosphate (0.025 M) (55 : 45, pH of 3.2). The flow rate was 1.0 mL/min and injection volume, 20 *μ*L. Quantitation was achieved by measurement of the peak area ratios of the drug to the internal standard. The retention time of the budesonide chromatographic peak was found at 5 min.

### 2.7. Stability Studies

Optimized formulation was kept in the humidity chamber maintained at 40°C and 75% relative humidity for 3 months. At the end of study, the formulation was evaluated for drug content and in vitro release profile. 

### 2.8. Statistical Analysis

The data of drug release were analyzed using one-way analysis of variance (ANOVA). The release profiles of optimized formulation were compared in stability and reproducibility tests using model-independent approach, with the similarity factor (*f*_2_) defined by [13]:

(1) $$f_{2}=50+\log\left\{{\left[1+\;\left(\frac{1}{n}\right)\overset{n}{\underset{t=1}{\sum }}n{\left(R_{t}-T_{t}\right)}^{2}\right]}^{-0.5}\times 100\right\}.$$

The two release profiles were considered to be similar if *f*_2_ value was more than 50 (between 50 and 100).

## 3. Results and Discussion

During this study, budesonide pellet core formulation was developed using extrusion-spheronization technique. These pellets were spherical in shape and showed suitable hardness to withstand coating conditions. The pellets had a 91 ± 2.83% budesonide release after 2 hrs in pH 6.8, so any later slow release could be attributed to the coating system(s) being studied. 

### 3.1. *In Vitro* Drug Release from Coated Pellets 

In designing an ideal colon-targeted drug delivery system, the drug should not be released in the stomach and small intestine, and the release of drug must be completed within the residence time of the dosage form in the colon. In the case of the present study, it was assumed that for colon-targeting purpose, an 18 h extended release formulation with a delay in onset of about 6 h would be suitable. This lag time would ensure the passage of the formulation intact through the stomach and small intestine without noticeable drug loss. 

The approach of using mixed polymeric coating of Eudragit NE 30D and Eudragit L30D-55 blends in time release applications has been reported previously [14]. Eudragit NE30D is an acrylic copolymer with neutral groups that enables controlled time release of the active ingredient by pH-independent swelling [5]. As its softening temperature is *ca. *12°C, it forms very flexible film suitable for compression with an elongation limit up to 300% due to the lack of strong interchain interaction [5]. Eudragit L30D-55 is an anionic polymer, which contains COOH as a functional group that dissolves at pH > 5.5. L30D-55 is known to be quite rigid with 20% elongation using 10% triethyl citrate as a plasticizer [15]. 

**Figure 2:**
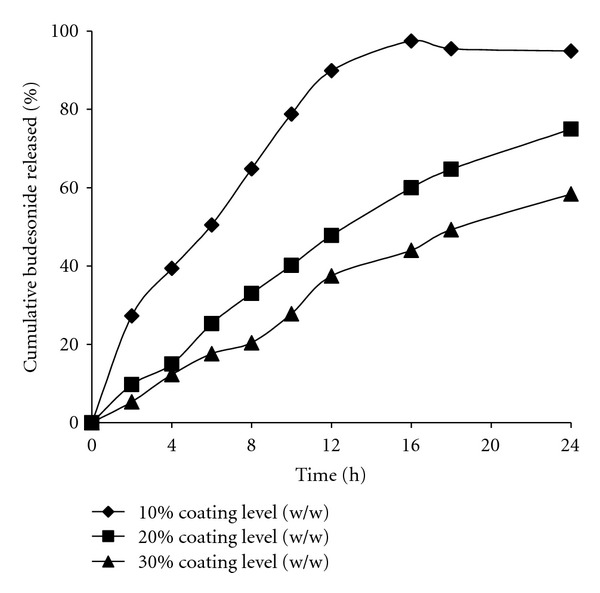
Effect of coating level of Eudragit NE 30D on budesonide release.

Four representative formulations of coated pellets were prepared by varying the ratio of Eudragit L to Eudragit NE as shown in Table 2. The results of *in vitro* drug release studies (Figure 2) indicated that increasing the polymer coating level of Eudragit NE30D from 15% to 30% (w/w) caused a significant reduction in the drug release. The pellets coated with Eudragit NE30D at a coating level of 30% (w/w) showed negligible release during the 6 h of dissolution test in HCl 0.1 N and PBS (pH 7.4). Nevertheless, at the end of dissolution studies, the mean percent drug released was only 58%. 

**Figure 3:**
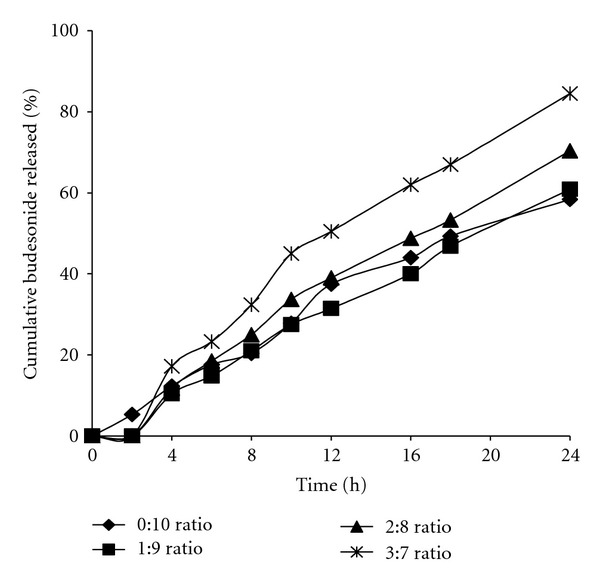
Effect of the ratio of Eudragit L 30D 55 to Eudragit NE 30D on budesonide release.

The effect of coating with Eudragit NE30D: Eudragit L30D-55 blend on *in vitro *drug release for three different batches of weight gains of 30% (w/w) is shown in Figure 3. Batches F_4_, F_5_, and F_6_ released no drug in acidic medium, 12.8%, 18.5%, and 23.3%, at the end of 6 hrs, while 57.4%, 70.5%, and 84.3% of drug was released at the end of 24 hrs, respectively. In PBS (pH 7.4), the enteric polymer (Eudragit L30D-55) dissolved or leached out, thus increasing the permeability of the coating, offering less resistance for budesonide diffusion. Although drug release of formulation F_6_ in simulating intestinal fluid was not optimal, the 3: 7 ratio of Eudragit L30D-55 to Eudragit NE30D was selected for further studies in consideration of the near complete release at the end of dissolution run. 

**Figure 4:**
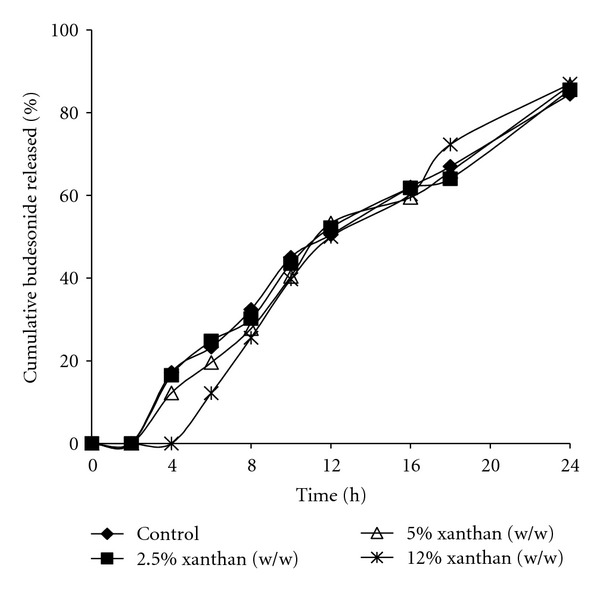
Budesonide release profiles from pellets with an inner coat of xanthan gum and an outer coat of Eudragit L 30D 55: Eudragit NE 30D (3 : 7 ratio) showing the effect of coating level of xanthan gum on budesonide release profile.

To achieve a desired release profile, a modification in the coating pattern was made. Xanthan gum as a release retardant polymer was chosen as the coating polymer for inner coating layer. Xanthan gum rapidly forms a gel layer that retards seeping of dissolution fluids into the core pellets and reduces the diffusion of drug from the core to negligible level and decreases the drug release from the formulation.  Figure 4 shows the release of budesonide from pellets coated with various coating levels of xanthan gum as inner coating. Coating with 2.5% (w/w) xanthan gum (F_7_) was not sufficient, and the drug release was the same as F_6_(*P* > 0.05). However, increasing the xanthan gum coating level to 12% (w/w) resulted in lower release in simulated intestinal fluid significantly (*P* < 0.05) with no effect on the total amount of drug released in 24 hrs. 

**Figure 5:**
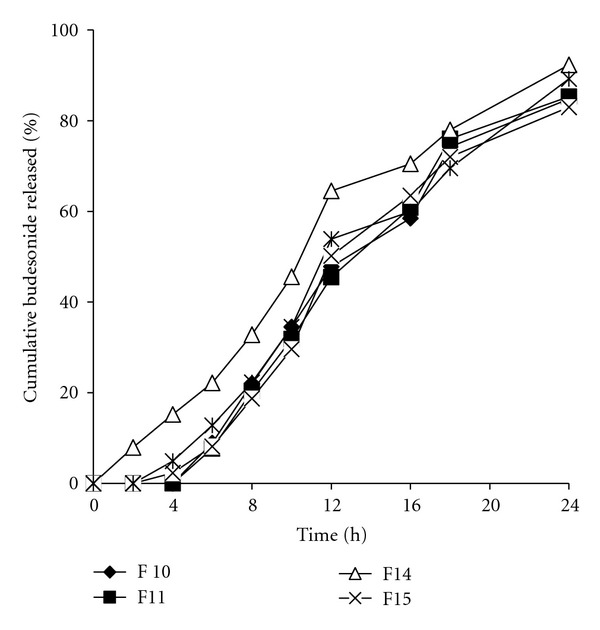
Release profile of selected coated pellets and multiunit tablets prepared with Cellactose in simulated GI fluid pH.

It seems that rapid swelling and rapid erosion properties of xanthan gum played an important role in this regard. By observing the above results, it was found that F_9_ batch released 12.16 ± 0.83% of the drug in the simulated intestinal fluid and released up to 84.54 ± 0.17% at the end of the 24 hrs in the simulated colonic medium considered as suitable batch for colon targeting. To further provids mechanical resistance and resistance against the influence of gastric juice, different coating thicknesses of Eudragit FS 30D were applied to budesonide pellets from 12 to 25% weight gain. Eudragit FS 30D is an anionic polymer of methacrylic acid and methacrylates that contains −COOH as a functional group and dissolves at pH 7. At the same time, FS 30D can achieve up to 300% elongation that enables decreased damage to the pellet coating during tabletting [16]. All Eudragit FS 30D coated pellets with 12%, as well as 20% (w/w) weight gain further suppressed budesonide release in simulated intestinal fluid but had no significant effect on total budesonide released at the end of dissolution run (Figure 5). As a thicker coating can prevent damage due to compression compared to the thinner coating and as the ability of pellets to undergo plastic and elastic deformation increases with increasing coating level [8], formulation F_11_ was selected to be combined with the inert tabletting granules in the preparation of multiple unit tablets.

### 3.2. *In Vitro* Drug Release from Tablets

A major problem in compaction of coated pellets is that the coating can rupture on compaction, resulting in significant differences in dissolution profiles of coated pellets prior to and after compaction. There are two approaches in pellet tabletting: tabletting of pellets without other excipients and tabletting of pellets together with pharmaceutically acceptable excipients. The approach of pellets compacting without other excipients does not have the problem of particle segregation, but formulation of pellet cores and also the coating of produced tablets is very difficult. Pellet cores must be deformable enough so that they form coherent tablets, and the coatings of pellets must be able to withstand compacting without damages, which can be ensured by formulating the coating of multiple units in such a way that the coating possesses improved elasticity. The approach of compacting of pellets together with tabletting excipients moderates requirements for the pellet coating elasticity, since plastically deformable tabletting excipients are able to partly absorb compaction forces and protect pellets from mechanical damages. This approach also enables easier obtaining of pellet-based tablets that have appropriate hardness and friability. To develop multiunit tablets of budesonide, coated pellets of F_11_ batch were mixed with Cellactose or Pearlitol granules as cushioning agents and compressed. The amount of cushioning agent was modified and studied for their effect on physical parameters of tablets and dissolution. Four tablet formulations were studied (F_12_, F_13_, F_14_ and F_15_) (Table 2) which were prepared at the same adjustment of press machine. Physical parameters of the tablets are shown in Table 3. All formulations were highly compressible resulting in tablets of enough crushing strength (Table 3). Friability of the tablets was also in the limits below 1% after 4 min of testing. But friability results were significantly lower with tablets F_14_ and F_15_. The results presented in Table 3 show that the content uniformity and average weight of F_12_ and F_13_ batches significantly changed during the tabletting. In contrast, the use of Cellactose produced tablets with improved content uniformity and average weight (F_14_ and F_15_). For these reasons F_12_ and F_13_ were excluded from further investigations. The *in vitro* drug release patterns of the F_14_ and F_15_ batches were compared and also compared to the pellets before compression as shown in Figure 5. In the case of batch F_14_, 7.96% of the drug was released after 2 hrs in gastric pH compared to negligible release from the pellets before compression. Then, the release became 14.32% after 4 hrs in phosphate buffer (pH 7.4), compared to 8.16% released from the pellets before compression. On the other hand, there was no difference in budesonide release from F_15_ and uncompressed pellets and the *f*_2_ value was 74.85. We conclude that the increasing concentration of Cellactose to 40% minimizes contact of multiple units with each other and protects the pellets from deformation under compression pressure. 

Recently a new technique has been introduced as MMX technology for production of colon-targeted tablets. Multimatrix (MMX) technology is a promising new delivery system that can improve efficacy of current and new drugs, augmenting targeting to the affected tract, thereby increasing response and remission rates for those drugs in patients with IBD. This technology comprises hydrophilic and lipophilic excipients, enclosed within a gastroresistant, pH-dependent coating of acrylic copolymers, which delay the release until the tablet reaches the indicated intestinal location where the programmed dissolution begins. The results of various studies involving MMX drugs have been published. Mesalamine MMX induces clinical and endoscopic remission in patients with mild-to-moderate ulcerative colitis (UC) compared with placebo. In a pilot study involving ten patients with UC, efficacy of heparin-MMX as an IBD therapy was observed. Positive results have also been observed with MMX budesonide 9 mg extended-release tablets in phase I studies [16]. Budesonide-MMX induced a fast and significant clinical improvement of active left-sided UC without suppression of adrenocortical functions and without important toxicity [17]. In comparison with Budesonide-MMX tablets, the designed pellet based tablets of our study contains 3 mg budesonide and is a pH- and time-controlled colon-targeted delivery system. In addition, tablets containing multiunits could be scored without losing the controlled release properties, which allows a more flexible dosing regimen and a more uniform spreading of the pellets through the colon.

### 3.3. Accelerated Stability Study 

Figure 5 shows the release profiles of optimized formulation (F_15_) at zero time and during storage period. No significant difference was found between the drug release profiles of the stored samples after three-month storage under accelerated conditions and *f*_2_ was 66.4. There were no signs of visually distinguishable changes in appearance and color of pellets. The drug content was comparable with that of the control samples and within limits (±10%). On the basis of these results, it can be concluded that the formulation had enough stability under accelerated stability test conditions for three months.

## 4. Conclusions

The study discusses the formulation of colon targeted multi unit tablets of budesonide for the treatment of UC. The pellets prepared for colon targeting of drug sufficiently protected drug release in the simulated environment of stomach as well as small intestine, and majority of drug release occurred in the simulated environment of colon. The budesonide-loaded pellets coated with 12% (w/w) xanthan gum, 30% (w/w) mixture of Eudragit NE: Eudragit L30D-55 (7: 3 ratio) and 25% (w/w) Eudragit FS 30D exhibited a promising dissolution profile. Cellactose granules as tabletting excipient, not only produced tablets with acceptable physical parameters, but also were able to protect the coated pellets from damage during tabletting and prevent premature drug release. The developed formulations were considered stable during 3 months of storage at accelerated stability conditions. Although the proposed formulation is moderately complex, its manufacture is simple and reproducible, and could also be easily manufactured on a large-scale in a reasonable processing time using standard pharmaceutical equipments. However, it should not be forgotten that the *in vitro* studies of the effects of pH and time on the release characteristics are really only a prelude to *in vivo* studies in human volunteers and then in patients with active ulcerative colitis. It should be considered that colonic pH changes in the presence of active inflammation, that small bowel transit usually slows with severe colitis, and that there is often stasis in the right colon in the presence of active distal disease. Thus, *in vivo* data are needed to really know whether the recommended formulation is going to be relevant. 

## Figures and Tables

**Table 1:** Operating conditions for the coating experiments.

Operating condition	Xanthan gum	Eudragit NE30D/L30D-55	Eudragit FS 30D
Before coating preheating to (°C)	—	—	—
Coating nozzle diameter (mm)	0.8	1.2	1.2
Spraying rate (g/min)	4	2	2
Inlet air temperature (°C)	60	30–32	35–42
Outlet air temperature (°C)	56-57	25–28	25–28
Curing on trays	24 hrs at 40°C	24 hrs at 40°C	24 hrs at 40°C

**Table 2:** Formulation details of pellets and multiunit tablets.

Formulation code	Dosage form	Pellet composition			Inert tabletting excipient (w/w% of tablet weight)
		Barrier coat Xanthan gum (%TWG)*	Inner coat L30D/NE30D (ratio) (%TWG)	Outer coat FS 30D (%TWG)	
F_1_	Coated pellets	—	(0 : 10) (10)	—	—
F_2_	Coated pellets	—	(0 : 10) (20)	—	—
F_3_	Coated pellets	—	(0 : 10) (30)	—	—
F_4_	Coated pellets	—	(1 : 9) (30)	—	—
F_5_	Coated pellets	—	(2 : 8) (30)	—	—
F_6_	Coated pellets	—	(3 : 7) (30)	—	—
F_7_	Coated pellets	2.5	(3 : 7) (30)	—	—
F_8_	Coated pellets	5	(3 : 7) (30)	—	—
F_9_	Coated pellets	10	(3 : 7) (30)	—	—
F_10_	Coated pellets	10	(3 : 7) (30)	12	—
F_11_	Coated pellets	10	(3 : 7) (30)	25	—
F_12_	Multiple unit tablet	10	(3 : 7) (30)	25	30 (Pearlitol)
F_13_	Multiple unit tablet	10	(3 : 7) (30)	25	50 (Pearlitol)
F_14_	Multiple unit tablet	10	(3 : 7) (30)	25	30 (Cellactose)
F_15_	Multiple unit tablet	10	(3 : 7) (30)	25	40 (Cellactose)

**Table 3:** Physical Characteristics of multiunit tablets of budesonide.

Formulation Code	Uniformity of weight [mg]	Hardness [*N*]	Friability [%]	Content uniformity
	RSD%			RSD%
F_12_	448 ± 12	68	0.72	2.917 ± 11.85
F_13_	622.9 ± 7.23	73	0.65	3.113 ± 7.29
F_14_	436.9 ± 4.28	64	0.14	3.046 ± 3.9
F_15_	513 ± 2.69	67	0.16	3.059 ± 1.85
